# Comparative assessment of dairy management practice adoption among SHG and non-SHG in tribal households of North Gujarat

**DOI:** 10.3389/fsoc.2026.1928015

**Published:** 2026-10-22

**Authors:** Khushboo Kumari, Laxmi R. Dubey, Dilip R. Vahoniya

**Affiliations:** 1 International Agribusiness Management Institute, Anand Agricultural University, Anand, Gujarat, India; 2 College of Agribusiness Management, Sardarkrushinagar Dantiwada Agricultural University, Banaskantha, Gujarat, India

**Keywords:** dairy farming, livestock livelihoods, rural development, tribal women, women’s empowerment

## Abstract

Dairy-based livelihoods provide an important source of income and employment for tribal households, with women playing a substantial role in livestock management. Self-Help Groups (SHGs) provide an institutional setting for collective savings, credit and livelihood support; however, differences in dairy management adoption and socioeconomic empowerment between SHG and non-SHG women require further empirical assessment. This study compared dairy management practice adoption, socioeconomic characteristics, empowerment and associated factors among tribal women in selected areas of the Sabarkantha and Aravalli districts of North Gujarat. An ex-post facto, cross-sectional research design was adopted. Multistage sampling was used to select 160 respondents, comprising 80 SHG members and 80 non-SHG members. Data were collected through structured personal interviews during 2024–2025. Adoption was assessed using a 35-item, three-point adoption index, while economic and social empowerment were measured using normalised indices. Welch’s independent-samples t-test, multiple linear regression and Garrett Ranking Technique (GRT) were used for analysis. The overall adoption score was significantly higher among SHG members (70.33 ± 13.83) than non-SHG members (58.45 ± 5.11). Significant differences were also observed in economic empowerment (65.49 ± 22.58 vs. 54.06 ± 21.21) and social empowerment (69.40 ± 8.92 vs. 37.77 ± 6.53). Landholding and herd size were statistically associated with adoption among SHG members, whereas dairy experience and annual income were associated with non-SHG members. Major constraints included high input costs, veterinary access, non-remunerative milk prices, credit availability and limited technical information. The findings indicate group-level differences in adoption and empowerment, while the cross-sectional and non-random design precludes causal interpretation of SHG membership.

## 1 Introduction

Dairy farming is an important component of rural livelihoods in India, providing household income, employment and nutritional support. In tribal areas of Gujarat, livestock rearing is closely integrated with smallholder farming systems and women contribute substantially to dairy activities such as feeding, milking, animal shed management and animal healthcare. Previous studies have documented the participation of tribal and rural women in dairy enterprises and the adoption of improved dairy management practices (Christie and Prasad Chebrolu, 2020; Felix et al., 2022; Jagadeesha, 2024). However, the adoption of recommended dairy practices may vary according to socio-economic characteristics and access to institutional resources. Self-Help Groups (SHGs) have emerged as an important institutional mechanism for improving women’s access to savings, credit, information and collective support. In livestock-based livelihoods, SHG participation provides an institutional platform through which women may access financial services, training, extension information and other livelihood-related resources. Previous studies have reported differences in dairy management practices and socio-economic conditions between SHG and non-SHG women (Rahman and Gupta, 2015; Chadda et al., 2022; Felix et al., 2022). However, SHG membership is not randomly assigned and SHG and non-SHG women may differ in education, landholding, income, dairy experience, herd size and other characteristics. Therefore, observed differences between the two groups need to be interpreted as group-level associations rather than causal effects of SHG participation.

The tribal context of North Gujarat provides an important setting for examining these issues. According to district-level data for 2024, Scheduled Tribes (ST) constituted 23.16 and 21.18% of the populations of Sabarkantha and Aravalli, respectively. SHG records for 2025 further indicate that ST members accounted for 25.90 and 20.53% of SHG membership in Sabarkantha and Aravalli, respectively. Within these districts, substantial concentrations of ST members among SHG members were observed in the selected talukas: Vijaynagar (12,871 of 16,561 SHG members), Khedbrahma (6,490 of 11,241), Bhiloda (13,785 of 22,675) and Meghraj (2,187 of 7,864). These figures indicate the substantial presence of tribal women within SHG structures in the selected areas and provide a contextual basis for examining dairy management practices among SHG and non-SHG women (JJM, 2024; NRLM, 2025). The adoption of improved dairy management practices is also associated with the socioeconomic circumstances of farm households. Education, landholding, dairy experience, herd size and income may be related to access to inputs, information, financial resources and livestock services required for improved dairy management (Hagone and Basunathe, 2015; Tamuli et al., 2021; Parmar et al., 2025). At the same time, adoption alone does not fully describe women’s socioeconomic position. Economic empowerment, self-confidence, social recognition, skills and access to public services represent additional dimensions relevant to understanding women’s participation in livelihood activities. The Resources-Agency-Achievement framework of Kabeer (1999) provides a useful conceptual basis for examining these dimensions by relating access to resources and the ability to exercise agency to valued outcomes. Despite increasing research on SHGs, dairy development and women’s empowerment, empirical evidence integrating dairy-management practice adoption, economic and social empowerment and socioeconomic characteristics among tribal women, while comparing SHG and non-SHG members within the same geographical context, remains limited. In particular, evidence from the tribal-dominated districts of Sabarkantha and Aravalli in North Gujarat that simultaneously examines adoption across major dairy-management domains, socioeconomic associations with adoption, economic and social empowerment, financial performance of SHGs and constraints faced by both SHG and non-SHG women is limited. A comparative assessment can therefore provide context-specific evidence on the observed differences and the socioeconomic characteristics associated with dairy-management adoption.

Therefore, the present study was undertaken among tribal women engaged in dairy farming in selected areas of Sabarkantha and Aravalli districts in North Gujarat. The study specifically aimed to: (i) examine the socio-economic characteristics of SHG and non-SHG members; (ii) assess and compare the extent of adoption of dairy management practices; (iii) examine the statistical associations between selected socio-economic characteristics and dairy management practice adoption; (iv) assess and compare economic and social empowerment; (v) assess the financial performance of the sampled SHGs; and (vi) identify and rank the major constraints faced by SHG and non-SHG members in dairy management. By integrating these dimensions, the study provides a comparative and context-specific assessment of dairy management adoption and socio-economic empowerment among tribal women in North Gujarat. The findings can provide evidence for understanding areas requiring greater attention in dairy management, institutional support and women’s socio-economic capabilities, while recognising the non-random and cross-sectional nature of SHG membership.

## 2 Materials and methods

To achieve the study’s objectives, a systematic methodology was followed, including site selection, sampling, data collection and data analysis.

### 2.1 Study area

The study was conducted in 2024–2025 in Sabarkantha (23.03°N, 73.07°E) and Aravalli (23.5°N, 73.1°E) districts of North Gujarat, located in the western region of India (Supplementary Figure S1). The region is predominantly agrarian, with dairy farming serving as a complementary livelihood activity for rural and tribal households, particularly for women. Agriculture and livestock rearing form the backbone of the local economy and tribal women actively participate in dairy-related activities such as feeding, milking, shed management and basic healthcare. The presence of active SHGs under livelihood promotion programmes and well-established dairy cooperatives such as Sabar Dairy provides institutional support for dairy development in the region (NRLM, 2025). These institutional structures provide access to credit, training, input services and milk marketing channels. The districts were therefore considered relevant for examining dairy management practice adoption, socio-economic empowerment, financial performance and constraints among tribal women in SHG and non-SHG households.

### 2.2 Research design and theoretical framework

An ex-post facto research design with a cross-sectional approach was adopted for the study (Kerlinger, 1973). This design was considered appropriate because the study examined existing differences and statistical associations between SHG and non-SHG members without manipulating SHG membership or any other study variable. Data were collected from the selected respondents during the 2024–25 study period. The study was guided by the Resources-Agency-Achievement framework proposed by Kabeer (1999), which conceptualises women’s empowerment as a process involving access to resources, the ability to exercise agency and the achievement of valued outcomes. In the present study, selected socio-economic characteristics, including education, landholding, income, dairy experience and herd size, were considered resource-related characteristics. SHG participation was considered an institutional context associated with access to financial, informational and social resources. Dairy management practice adoption and economic and social empowerment were considered outcome dimensions. The framework provided a theoretical basis for interpreting the observed differences and statistical associations between SHG and non-SHG members and was not intended to establish a causal effect of SHG participation.

### 2.3 Sampling procedure

A multistage sampling procedure was adopted for the selection of respondents. In the first stage, Sabarkantha and Aravalli districts were purposively selected because they recorded the highest proportions of ST members among SHG members (25.90 and 20.53%, respectively) among the North Gujarat districts (NRLM, 2025). In the second stage, Vijaynagar (12,871) and Khedbrahma (6,490) talukas of Sabarkantha district and Bhiloda (13,785) and Meghraj (2,187) talukas of Aravalli district were purposively selected based on the number of ST members among SHG members and the relevance of dairy-based livelihoods (JJM, 2024; NRLM, 2025). These four talukas represented the areas with comparatively high concentrations of ST-SHG membership within the two selected districts. In the third stage, four villages from each selected taluka were randomly selected from the eligible villages, resulting in a total of 16 villages. In the fourth stage, eligible tribal women engaged in dairy farming were identified through local enquiry in each selected village. From among the available eligible respondents, five SHG members and five non-SHG members were selected from each village. Thus, the final sample consisted of 160 respondents, including 80 SHG members and 80 non-SHG members (Supplementary Table S1).

The sample size of 160 respondents was determined considering the scope of the study, availability of eligible respondents and the feasibility of conducting detailed face-to-face interviews within the available time and field resources. An *a priori* statistical power calculation was not performed. Equal allocation of respondents between the two groups was deliberately adopted to facilitate a balanced comparison of SHG members and non-SHG members. This equal allocation did not constitute formal matching based on age, education, landholding, herd size, income, or other socio-economic characteristics. The districts and talukas were purposively selected; therefore, the sample was not intended to be statistically representative of all tribal women or dairy farmers in Gujarat. The findings should consequently be interpreted within the context of the selected study areas and generalisation to other regions should be made with caution. The sample size and village-level allocation were also comparable with those reported in other agricultural extension and rural livelihood studies. Primary data were collected through personal interviews using a structured and pre-tested interview schedule designed to obtain information on socio-economic characteristics, dairy management practices, empowerment dimensions, financial performance and constraints (Supplementary Tables S2–S7). Secondary information was obtained from relevant SHG records, cooperative reports and government publications.

### 2.4 The socio-economic profile of tribal women of SHG members and non-SHG members

The socio-economic profiles of tribal women who were SHG members and non-SHG members were examined using ten key parameters: age, education, family size, landholding, family occupation, housing type, dairy experience, herd size, milk yield and annual income. Respondents were categorised using standardised classifications adopted from previous studies, (Manjunath, 2014; NAEP, 2023; Pandya and Pandya, 2008; Raval and Chandwat, 2011; Savita, 2004; Sowjanya, 2014), as presented in Supplementary Table S2. Frequency and percentage were used to summarise the socio-economic characteristics of the two groups. To examine the adjusted associations between selected socio-economic characteristics and dairy management practice adoption, multiple linear regression analysis was performed separately for SHG members and non-SHG members (*n* = 80 in each group). The total dairy management adoption score was considered the dependent variable (Y), while the ten selected socio-economic characteristics were considered explanatory variables (X). The regression coefficient (*B*), standardised beta coefficient (*β*), *t*-value and *p*-value were used to assess the individual associations of the explanatory variables with the adoption score, while *R*^2^, adjusted *R*^2^ and the *F*-test were used to assess the overall model fit. Categorical explanatory variables were appropriately coded before inclusion in the regression model. The analysis was used to identify socio-economic characteristics that were statistically associated with dairy management practice adoption after accounting for other variables included in the model. The regression model used in the analysis is expressed in Equation 1:

$$Y=a+\sum b_{i}X_{i}+e$$ (1)

where Y represents the total dairy management adoption score, a is the intercept, b_i_ represents the regression coefficient of the respective explanatory variable, X_i_ represents socio-economic characteristics and e represents the random error term.

### 2.5 Extent of adoption of dairy management practices

The dairy management practice adoption index was developed through a systematic identification and refinement of recommended dairy management practices. Initially, relevant literature and published studies (Meena et al., 2013; Rahman and Gupta, 2015; Felix et al., 2022), particularly those related to dairy farming and tribal communities, were reviewed to identify commonly recommended and contextually relevant dairy management practices. The preliminary list of practices was subsequently discussed with livestock management and dairy specialists at Sardarkrushinagar Dantiwada Agricultural University (SDAU) to assess their relevance, clarity and applicability to the study area. Based on the literature review, expert consultation and field relevance, the practices were finalised and organised into major management domains. The content relevance and applicability of the items were assessed through consultation with livestock and dairy specialists, while the interview schedule was pre-tested before the final survey to identify ambiguity and improve the clarity of the questions. Necessary modifications were incorporated before the final data collection. A final set of 35 practices, presented in Supplementary Table S3, was included in the adoption index, namely breeding (5), feeding (11), animal shed management (5), healthcare (6), milking (4) and other management practices (4). Each of the 35 practices was assigned equal weight because the objective was to assess the overall extent of adoption across the selected dairy management practices rather than to assign subjective differential importance to individual practices.

The extent of adoption was measured among 160 tribal women (80 SHG members and 80 non-SHG members) using a three-point ordinal scale: fully adopted (3), partially adopted (2) and not adopted (1). Full adoption indicated that the recommended practice was followed completely and regularly according to the specified recommendation; partial adoption indicated that the practice was followed incompletely, irregularly, or that only some components of the recommended practice were followed; and non-adoption indicated that the recommended practice was not followed. The practice-specific operational criteria used to distinguish full, partial and non-adoption are provided in Supplementary Table S3. Since a score of 1 was assigned to non-adoption, the theoretical total adoption score ranged from 35 to 105, where a score of 35 represented non-adoption of all the selected practices and did not indicate adoption of 35 practices. The total adoption score for each respondent was obtained by summing the scores assigned to all 35 practices. Based on the mean (X̄) and standard deviation (SD) of the total adoption score, respondents were classified into three categories: low adoption (less than X̅ − S.D.), medium adoption (between X̅ ± S.D.) and high adoption (more than X̅ + S.D.). This classification was used to categorise respondents according to their overall adoption level and to compare adoption patterns between SHG members and non-SHG members.

### 2.6 Socio-economic empowerment index

The socio-economic empowerment index was developed to assess and compare the economic and social empowerment of SHG and non-SHG members, following the methodology of Singh and Singh (2014). Two separate indices were constructed: the economic empowerment index (En) and the social empowerment index (Sn), calculated using Equations 2, 3 respectively. The indicators, scoring criteria and maximum attainable scores are provided in Supplementary Tables S5–S7. The economic empowerment index comprised six indicators: household assets, household income, expenditure, savings, loan amount and housing type. Each indicator was scored according to the specified criteria and expressed relative to its maximum attainable score. The normalised indicator scores were combined using equal weighting to obtain the economic empowerment index (En). Similarly, the social empowerment index comprised four dimensions: self-confidence, social awareness and recognition, skills and access to public facilities/services. Each dimension was scored using the specified criteria, normalised relative to its maximum attainable score and combined using equal weighting to obtain the social empowerment index (Sn). Higher index values represented higher levels of economic or social empowerment. The theoretical minimum and maximum values of the indices were determined from the scoring framework.

$$\text{Economic Index}\;(\mathrm{En})=\sum E_{i}/\sum E_{i(\max)}$$ (2)

$$\text{Social Index}\;(\mathrm{Sn})=\sum S_{i}/\sum S_{i(\max)}$$ (3)

Where, E_i_/S_i_ represents the score obtained for the i^th^ economic/social indicator, E_i(max)_/S_i(max)_ represents the maximum attainable score for the corresponding i^th^ economic/social indicator.

### 2.7 Financial performance of SHG

The financial performance of SHGs was assessed using four indicators: recovery ratio, thrift-credit ratio, outstanding-credit ratio and savings amount. The recovery ratio, thrift-credit ratio and outstanding-credit ratio were calculated using the respective formulae described by Srinivasan et al. (2001), as presented in Equations 4–6, respectively. The average values of these financial indicators were used to assess the financial performance of the SHGs. The savings amount was recorded as the amount of savings reported/maintained by the respondents or SHGs during the 2024–2025 study period. The required financial information was obtained from relevant SHG records and respondent-level information for the reference period considered in the study. The formulae used to calculate the financial performance indicators are presented below:

$$\begin{array}{l} \text{Average Recovery Ratio}=\\ \frac{\text{Actual amount recovered during the time period}}{\text{Total demand raised during the time period}}\;x\;100 \end{array}$$ (4)

$$\text{Thrift Credit Ratio}=\frac{\text{Total saving}\;}{\text{Total lending}}$$ (5)

$$\text{Outstanding Credit Ratio}=\frac{\text{Total outstanding amount}\;}{\text{Total lending amount}}$$ (6)

### 2.8 Constraints faced by tribal women in livestock production

Constraints in livestock production were operationally defined as the difficulties and limitations encountered by tribal women while carrying out various dairy management activities, including breeding, feeding, healthcare, milking and other related activities. The constraints were identified through a procedure similar to that used for selecting dairy management practices and were finalised after consultation with relevant subject-matter experts. The finalised list of constraints is presented in Table 1. To prioritise the constraints according to their perceived importance, the Garrett Ranking Technique (GRT) was employed following Garrett and Woodworth (1969). Respondents were asked to rank the identified constraints according to their perceived severity or importance. The ranks assigned by each respondent were converted into percentage positions using Equation 7. The obtained percentage positions were converted into Garrett scores using the standard Garrett Ranking Table. The scores for each constraint were summed across respondents and the mean Garrett score was calculated. The constraints were subsequently ranked according to their mean Garrett scores, with a higher mean Garrett score indicating greater perceived severity or importance.

$$\text{Percent Position}=\frac{\left[100(R_{\mathrm{ij}}-0.05)\right]}{N_{j}}$$ (7)

**Table 1:** Constraints faced by tribal women who were SHG members and non-SHG members in the adoption of dairy management practices.

Sr. No.	Particulars	Members (*n* = 80)		Non-SHG members (*n* = 80)	
		Average Garret score	Rank	Average Garret score	Rank
I	Constraints related to breeding management practices				
1	Non-availability of good crossbred cows/ improved buffaloes in local markets	54.99	II	55.05	II
2	High cost of improved animal breeds	62.36	I	61.21	I
3	Lack of knowledge about Artificial Insemination (AI)	44.03	III	45.18	III
4	Difficulty in heat detection	38.63	IV	38.56	IV
II	Constraints related to feeding management practices				
1	Lack of availability of green fodder in the summer season	54.08	II	64.58	I
2	High cost of concentrates /mineral mixture	64.00	I	53.08	II
3	Lack of knowledge about the enrichment of dry fodder	44.73	III	38.50	IV
4	Inadequate knowledge about balanced ration and proper feeding of that	37.20	IV	43.85	III
III	Constraints related to healthcare management practices				
1	Distant location of veterinary hospitals	62.00	I	59.56	II
2	High cost of medicines	61.38	II	52.13	III
3	Lack of veterinary services on time	50.50	III	45.00	IV
4	Lack of information about vaccination programmes	42.75	IV	36.31	V
5	Lack of knowledge about common contagious diseases, their causes and control measures	33.38	V	57.00	I
IV	Constraints related to milking management practices				
1	Non-remunerative price of the milk	65.44	I	63.06	I
2	Lack of knowledge in making value-added dairy products (Panner, Khova, Cheese *etc*.)	36.94	III	47.63	II
3	The non-existence of milk producer cooperative societies at the village level	47.63	II	39.31	III
V	General constraints				
1	Non-availability of sufficient loans	64.49	I	50.24	II
2	Lack of information about dairy development schemes of the government	46.68	II	40.26	III
3	Lack of knowledge about animal insurance	38.84	III	59.50	I

Where, R_ij_ = Rank allotted for i^th^ problem by the j^th^ individual, N_j_ = Number of problems ranked by the j^th^ individual.

### 2.9 Statistical tools and analytical techniques

The collected data were analysed using appropriate descriptive and inferential statistical techniques in accordance with the study objectives. Frequency and percentage were used to summarise the socio-economic characteristics of respondents, adoption levels and empowerment categories. X̄ and SD were used to describe continuous variables and index scores, as presented in Equations 8, 9, respectively. The Welch independent-samples t-test was used to compare the mean adoption and empowerment scores between SHG members and non-SHG members, as presented in Equation 10. Multiple linear regression analysis was used to examine the statistical associations between selected socio-economic characteristics and dairy management practice adoption, separately for SHG members and non-SHG members. The GRT was used to prioritise the constraints faced by respondents according to their perceived severity or importance. Statistical analyses were interpreted in accordance with the study objectives and a two-tailed *p*-value < 0.05 was considered statistically significant.

$$\bar{X}=\frac{\sum X_{i}}{n}$$ (8)

$$\mathrm{SD}=\sqrt{\frac{\sum {\left(X_{i}-\bar{X}\right)}^{2}}{n-1}}$$ (9)

$$t=\frac{{\bar{X}}_{1}-{\bar{X}}_{2}}{\sqrt{\;\frac{s_{1}^{2}}{n_{1}}+\frac{s_{2}^{2}}{n_{2}}}}$$ (10)

Where, $\sum X_{i}$ = Observed value, n = Number of respondents, X_1_ = mean score of SHG members, X_2_ = mean score of non-SHG members, s^2^_1_ = variance of SHG members, s^2^_2_ = variance of non-SHG members, n_1_ = number of SHG members and n_2_ = number of non-SHG members.

## 3 Results and discussion

### 3.1 Socio-economic profile of respondents

The socio-economic profiles of SHG and non-SHG members differed across the characteristics examined (Supplementary Figure S2). SHG members had higher proportions of respondents with secondary and higher-secondary education, larger landholdings, pucca housing, longer dairy experience, larger herd sizes, higher milk yield and higher annual family income. Secondary education was reported by 37.50% of SHG members compared with 18.75% of non-SHG members, while 33.75% of SHG members reported annual family income above ₹1,20,000 compared with 10.00% of non-SHG members. Similarly, 53.75% of SHG members had more than 10 years of dairy experience compared with 31.25% of non-SHG members and 36.25% had large herd sizes compared with 15.00% of non-SHG members. These findings indicate notable socio-economic and dairy-farming differences between the two groups.

Multiple linear regression was used to examine the adjusted associations between selected socioeconomic characteristics and dairy management adoption separately among SHG and non-SHG members (Table 2). The regression models for SHG and non-SHG members are presented in Equations 11, 12, respectively. Among SHG members, landholding (*B* = 3.007, *p* = 0.022) and herd size (*B* = 11.061, *p* = 0.002) were positively associated with adoption. The regression model explained 41.7% of the variation in adoption (*R*^2^ = 0.417; adjusted *R*^2^ = 0.332; *F* = 4.930, *p* < 0.001). Among non-SHG members, dairy experience (*B* = 1.995, *p* = 0.001) and annual income (*B* = 1.528, *p* = 0.046) were positively associated with adoption, while the model explained 36.3% of the variation (*R*^2^ = 0.363; adjusted *R*^2^ = 0.271; *F* = 3.934, *p* < 0.001). Overall, the regression results indicate that different socioeconomic characteristics were statistically associated with dairy management adoption within the two groups. These associations should be interpreted cautiously because SHG membership was not randomly assigned and the groups were not formally matched. Therefore, the observed differences cannot be interpreted as causal effects of SHG membership.

**Table 2:** Multiple regression analysis of socio-economic factors associated with dairy management adoption.

Socio-economic variable	SHG members			Non-SHG members		
	*B*	*β*	*t*	*B*	*β*	*t*
Age	2.007	0.132	1.378	1.464	0.197	1.963
Education	2.683	0.199	1.949	0.792	0.143	1.397
Family size	−0.53	−0.027	−0.261	−0.277	−0.033	−0.328
Housing type	2.152	0.115	1.194	0.222	0.031	0.309
Landholding	3.007	0.224	2.342*	1.23	0.152	1.41
Family occupation	1.211	0.05	0.44	−0.437	−0.047	−0.429
Dairy experience	−0.121	−0.007	−0.056	1.995	0.355	3.43**
Herd size	11.061	0.561	3.278**	1.755	0.224	1.563
Milk yield	−5.608	−0.294	−1.532	0.924	0.113	0.809
Annual income	4.2	0.209	1.548	1.528	0.212	2.032*
*R* ^2^	0.417			0.363		
Adjusted *R*^2^	0.332			0.271		

*B*, unstandardised regression coefficient; *β*, standardised regression coefficient; *t*, regression coefficient t-statistic; Significant at *p < 0.05; **p < 0.01.

SHG members:

$$\begin{array}{l} Y=21.844+3.007X_{1}+2.683X_{2}-0.530X_{3}\\ +2.152X_{4}+3.007X_{5}+1.211X_{6}-0.121X_{7}\\ +11.061X_{8}-5.608X_{9}+4.200X_{10} \end{array}$$ (11)

Non-SHG members:

$$\begin{array}{l} Y=41.046+1.464X_{1}+0.792X_{2}-0.277X_{3}\\ +0.222X_{4}+1.230X_{5}-0.437X_{6}+1.995X_{7}\\ +1.755X_{8}+0.924X_{9}+1.528X_{10} \end{array}$$ (12)

where Y represents the dairy management adoption score, X_1,_ age; X_2,_ education; X_3,_ family size; X_4,_ housing type; X_5,_ landholding; X_6,_ family occupation; X_7,_ dairy experience; X_8,_ herd size; X_9_, milk yield and X_10_, annual income. The term e represents the error term or the unexplained variation in the dairy management adoption score.

### 3.2 Extent of adoption of dairy management practices by tribal women SHG members and non-SHG members

#### 3.2.1 Extent of adoption of breeding management practices

The adoption of breeding management practices varied between SHG and non-SHG members (Figure 1). The non-adoption of improved breeds was reported by 56.25 and 58.75% of SHG and non-SHG members, respectively, indicating a common gap in this practice. Regular monitoring of oestrous symptoms was reported by 43.75% of SHG members compared with 35.0% of non-SHG members. Similarly, the adoption of timely breeding within 12–14 h of heat symptoms was 22.5% among SHG members, compared with only 3.75% among non-SHG members. However, the overall breeding management adoption score did not differ significantly between the groups (*p* = 0.379; Table 3). Thus, while differences were observed for some individual practices, the aggregate level of breeding management adoption was statistically comparable between the two groups. Similar observations were reported by Kumar (2016) and Felix et al. (2022).

**Figure 1:**
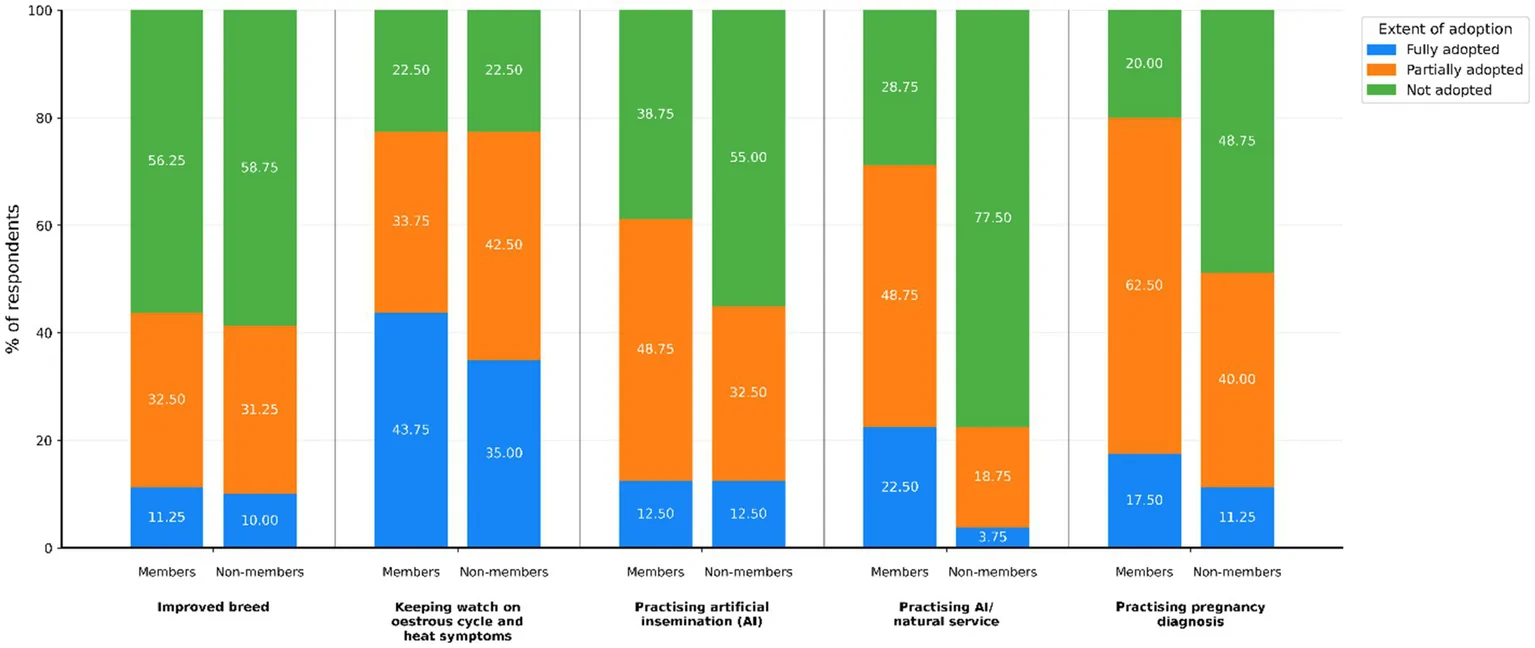
Distribution of respondents based on their extent of adoption in breeding management practices.

**Table 3:** Comparison of dairy management practice adoption across major management components among SHG and non-SHG members.

S. No.	Component	SHG X̅ ± SD	Non-SHG X̅ ± SD	t
1.	Breeding management	8.90 ± 2.46	8.61 ± 1.56	0.88
2.	Feeding management	22.45 ± 5.93	17.15 ± 2.93	7.17**
3.	Animal shed management	10.00 ± 3.18	8.49 ± 1.59	3.81**
4.	Healthcare management	10.53 ± 3.31	9.45 ± 1.69	2.59*
5.	Milking management	10.40 ± 2.73	8.01 ± 1.82	6.52**
6.	Other management	8.05 ± 1.83	6.74 ± 1.47	4.99**
7	Overall adoption score	70.33 ± 13.83	58.45 ± 5.11	7.21**

t, Welch independent-samples *t*-test; Significant at *p < 0.05; **p < 0.01.

#### 3.2.2 Extent of adoption of feeding management practices

The adoption of feeding management practices was generally higher among SHG members than among non-SHG members (Figure 2). Full adoption of green fodder cultivation was reported by 22.5% of SHG members, compared with 6.25% of non-SHG members. A notable difference was also observed in proper colostrum feeding, with full adoption reported by 30% of SHG members compared with 6.25% of non-SHG members. Similarly, 30% of SHG members reported full adoption of providing clean drinking water, compared with 12.5% of non-SHG members. Overall, the feeding management adoption score was significantly higher among SHG members than non-SHG members (Table 3). These findings are consistent with those of Keerthi et al. (2022) and Bariya et al. (2022), who reported variation in the adoption of improved feeding and fodder management practices among dairy farmers.

**Figure 2:**
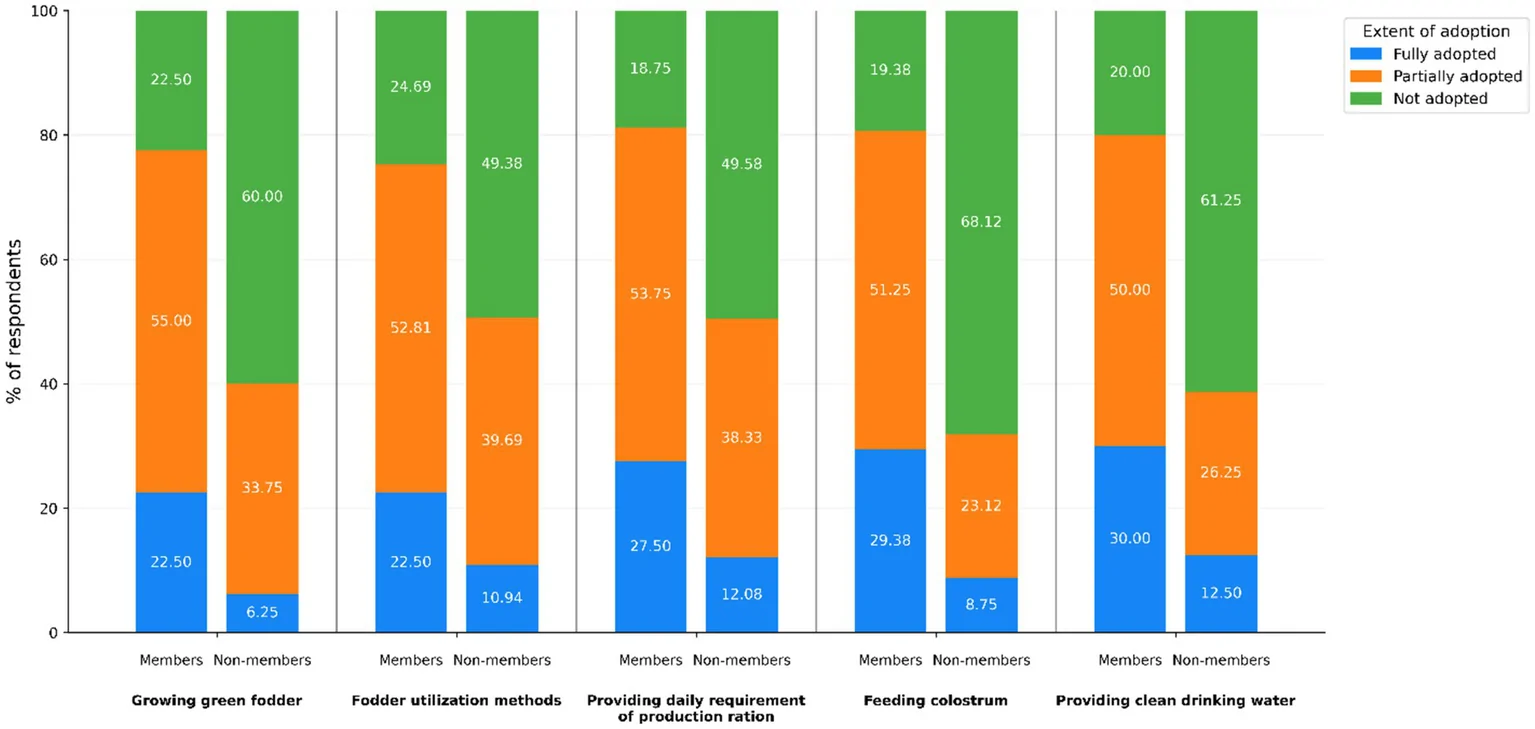
Distribution of respondents based on their extent of adoption in feeding management practices.

#### 3.2.3 Extent of adoption of animal shed management practices

The adoption of animal shed management practices was higher among SHG members than among non-SHG members (Figure 3). Full adoption of the recommended head-to-head or tail-to-tail housing system was reported by 26.25% of SHG members, compared with 8.75% of non-SHG members. Similarly, full adoption of an ideal byre with appropriate height, slope and drainage was reported by 30% of SHG members, compared with 16.25% of non-SHG members. SHG members also showed a comparatively higher adoption of practices related to calf housing, ventilation, lighting and shed cleanliness. Overall, the animal shed management adoption score was significantly higher among SHG members than non-SHG members (Table 3). Similar patterns have been reported by Rahman and Gupta (2015), Kumar (2016), and Felix et al. (2022), who documented differences in the adoption of improved animal housing and hygiene practices among dairy farmers.

**Figure 3:**
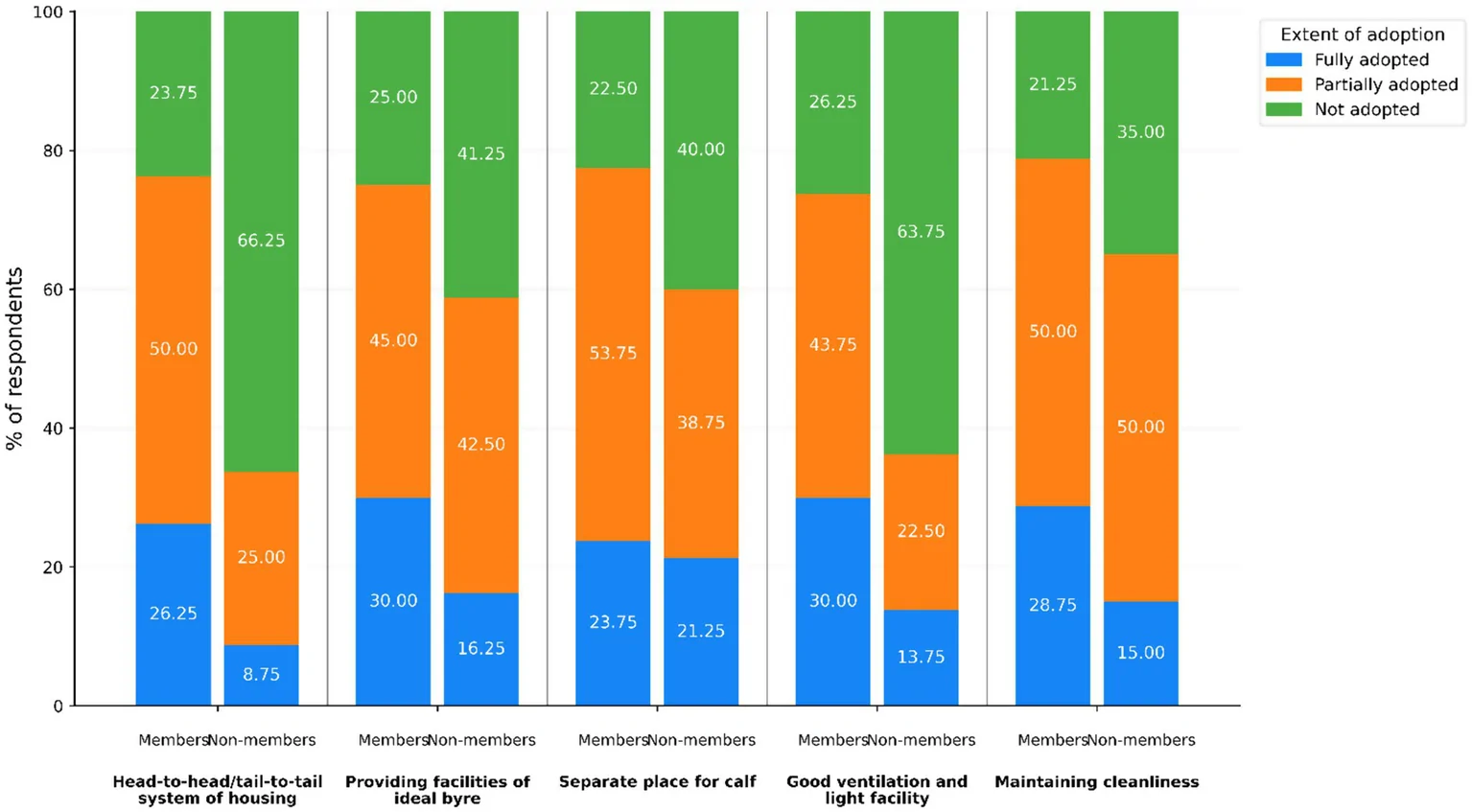
Distribution of respondents based on their extent of adoption in animal shed management practices.

#### 3.2.4 Extent of adoption of healthcare management practices

The adoption of healthcare management practices was generally higher among SHG members than among non-SHG members (Figure 4). Full adoption of daily grooming and bathing of animals was reported by 28.75% of SHG members, compared with only 3.75% of non-SHG members. Similarly, 62.5% of SHG members adopted mucus removal from newborn calves, compared with 30% of non-SHG members. A considerable proportion of non-SHG members (61.25%) reported non-adoption of deworming. Timely vaccination also showed relatively low full adoption in both groups, at 15 and 11.25% among SHG and non-SHG members, respectively. Overall, the healthcare management adoption score was significantly higher among SHG members than among non-SHG members (Table 3). Similar differences in the adoption of dairy healthcare practices have been reported by Rahman and Gupta (2015) and Felix et al. (2022).

**Figure 4:**
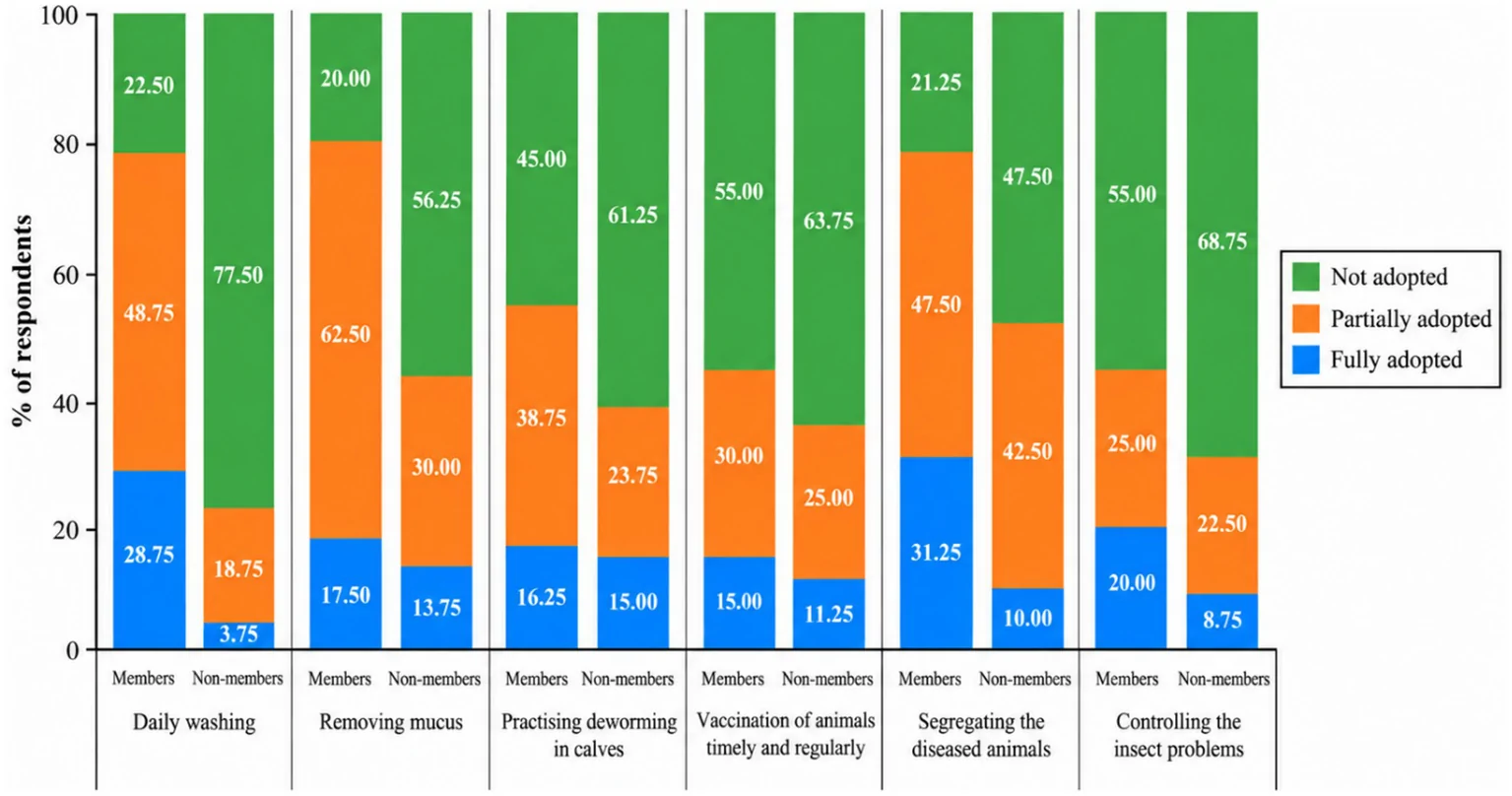
Distribution of respondents based on their extent of adoption in healthcare management practices.

#### 3.2.5 Extent of adoption of milking management practices

The adoption of milking management practices was higher among SHG members than non-SHG members (Figure 5). Full adoption of the full-hand milking method was reported by 28.75% of SHG members, compared with 10% of non-SHG members. Similarly, the use of clean and washed utensils was fully adopted by 26.25% of SHG members compared with 12.5% of non-SHG members, while milk filtering was adopted by 23.75 and 7.5%, respectively. Maintenance of milk sale records was also more common among SHG members (25%) than non-SHG members (11.25%). Overall, the milking management adoption score was significantly higher among SHG members than non-SHG members (Table 3). Similar differences in the adoption of hygienic milking and record-keeping practices have been reported by Rahman and Gupta (2015), Chadda et al. (2022), Kumar (2016), and Felix et al. (2022).

**Figure 5:**
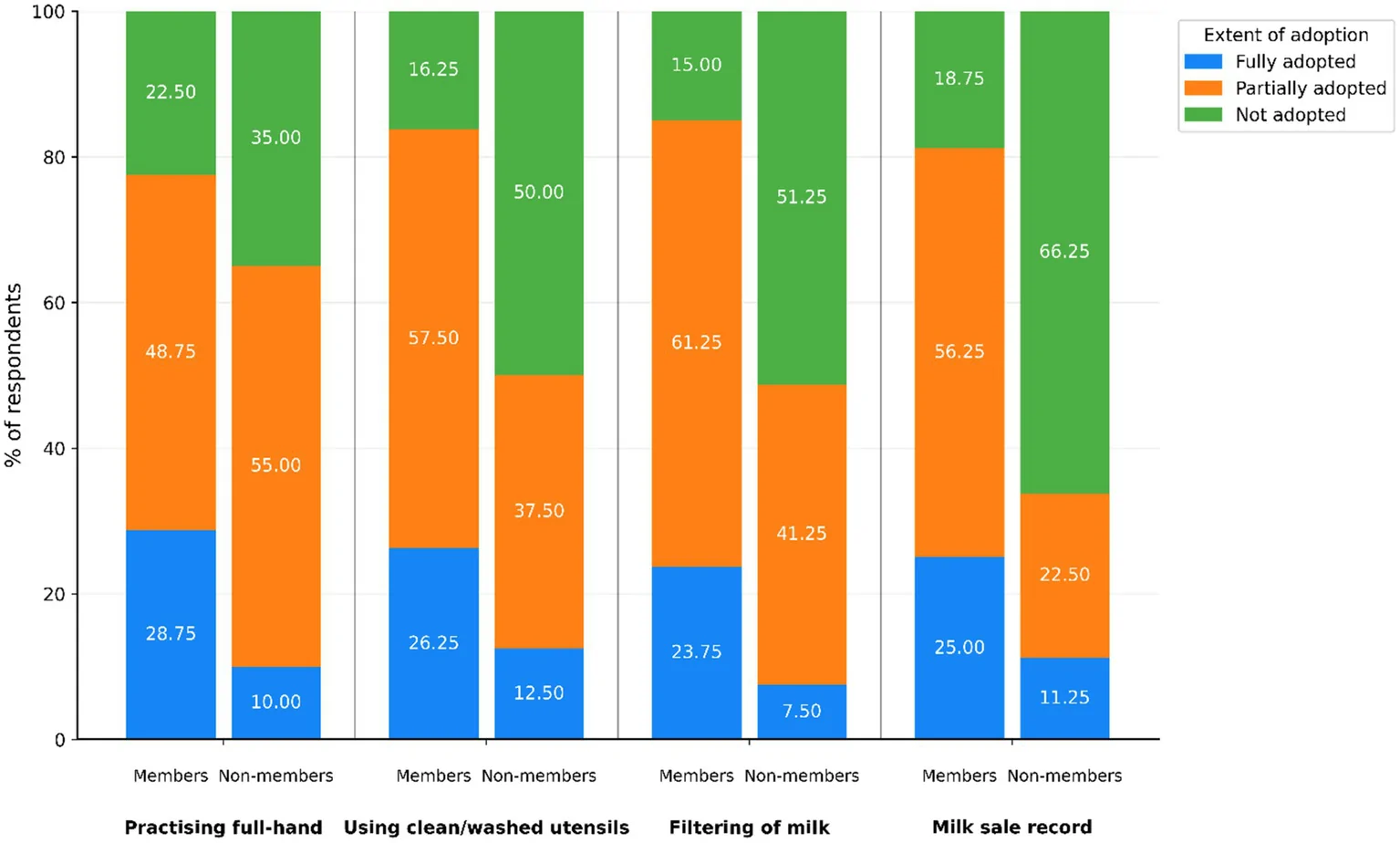
Distribution of respondents based on their extent of adoption in milking management practices.

#### 3.2.6 Extent of adoption of other management practices

The adoption of other dairy management practices was generally higher among SHG members than among non-SHG members (Figure 6). Full adoption of controlling undesirable animal behaviour was reported by 23.75% of SHG members, compared with 8.75% of non-SHG members. The practice of maintaining an appropriate dry period before parturition also showed higher adoption among SHG members, with 17.5% reporting full adoption compared with 11.25% of non-SHG members. Similarly, full adoption of dairy record-keeping was reported by 22.5% of SHG members and 18.75% of non-SHG members, while animal insurance was reported by 31.25 and 25%, respectively. Overall, the adoption score for other management practices was significantly higher among SHG members than non-SHG members (Table 3). Similar variation in the adoption of preventive and general dairy management practices was reported by Felix et al. (2022).

**Figure 6:**
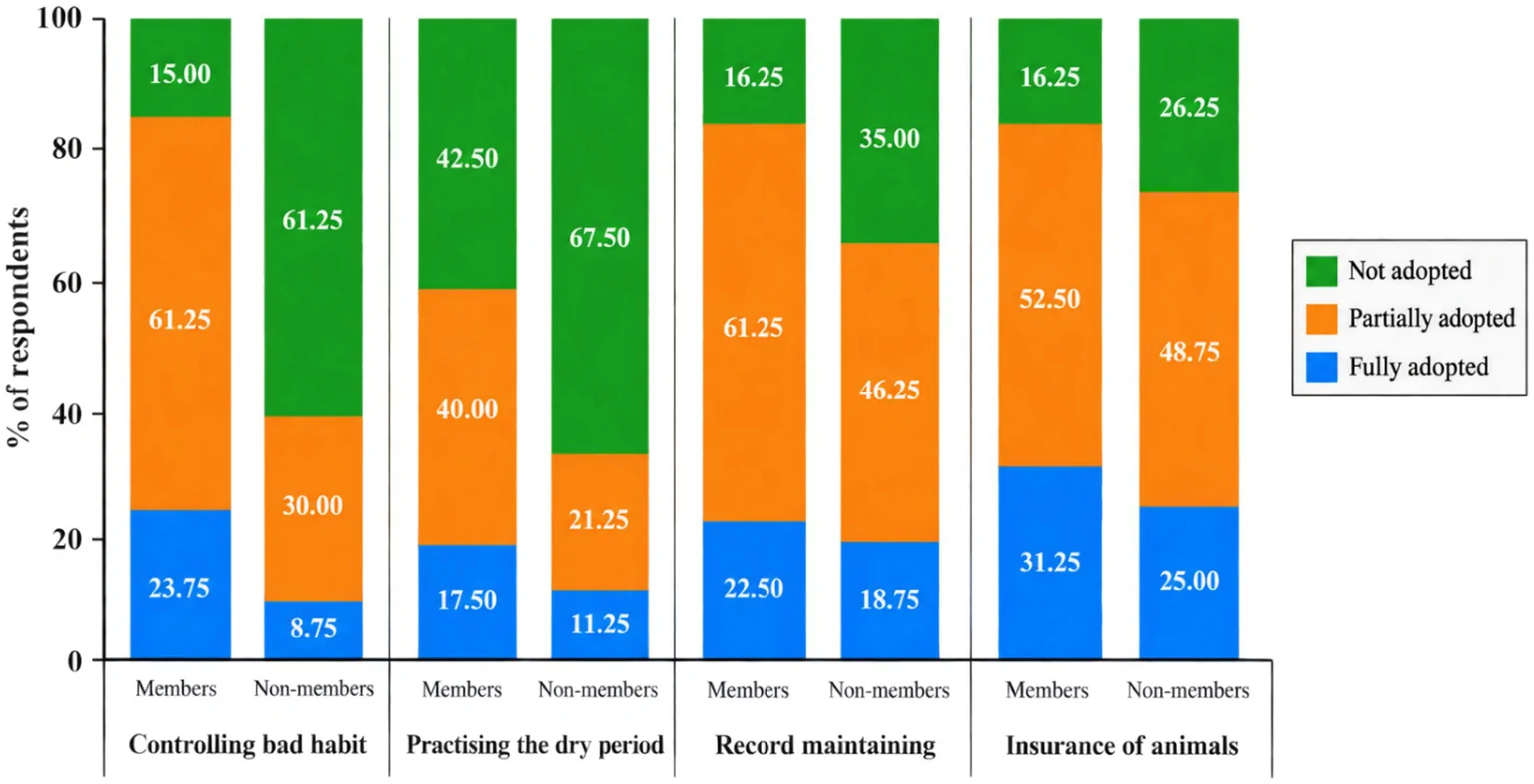
Distribution of respondents based on their extent of adoption in other management practices.

#### 3.2.7 Overall extent of adoption of dairy management practices by tribal women SHG members and non-SHG members

The overall extent of dairy management practice adoption differed between SHG and non-SHG members. High adoption was reported by 35% of SHG members compared to only 5% of non-SHG members, whereas the majority of non-SHG members (83.75%) were in the medium-adoption category. Among SHG members, 56.25% were in the medium and 8.75% in the low-adoption category (Supplementary Table S4). This distribution indicates that although medium adoption was common in both groups, a larger proportion of SHG members was represented in the high-adoption category. The component-wise comparison showed higher mean adoption scores among SHG members across all six dairy management components (Table 3). Differences were highly significant (*p* < 0.01) for feeding, animal shed, milking and other management practices, while healthcare management showed a significant difference (*p* < 0.05). In contrast, breeding management did not differ significantly between the groups (*p* = 0.379). The overall adoption score also differed significantly between SHG and non-SHG members (*p* < 0.01). The comparatively larger differences in feeding and milking practices suggest that these areas contributed substantially to the observed variation in overall adoption.

Similar differences in the adoption of improved dairy management practices among women associated with SHGs have been reported by Keerthi et al. (2022), Bariya et al. (2022), and Felix et al. (2022). These studies reported variation in the adoption of improved feeding, housing, healthcare and other dairy management practices among women associated with group-based programs. The consistency of these findings with the present study provides contextual support for the observed group differences. However, because the present study used a cross-sectional design and SHG membership was not randomly assigned, these differences should be interpreted as group-level associations rather than evidence of a causal effect of SHG membership.

### 3.3 Socio-economic empowerment index

#### 3.3.1 Economic empowerment index

The economic empowerment indicators showed differences between SHG and non-SHG members (Supplementary Tables S5, S6). For example, 31.25% of SHG members had household assets above ₹72,500, compared with 13.75% of non-SHG members. Similarly, savings above ₹9,800 were reported by 35% of SHG members, compared with 15% of non-SHG members. A difference was also observed in housing status, with 47.5% of SHG members living in pucca-owned houses, compared with 33.75% of non-SHG members. The mean economic empowerment index score was 65.49 ± 22.58 for SHG members and 54.06 ± 21.21 for non-SHG members, with the difference being statistically significant (*p* < 0.01) (Table 4). These findings indicate a measurable difference in the economic empowerment scores of the two groups. Similar differences in economic empowerment among women participating in SHGs have been reported by Kumar and Rao (2021). However, given the cross-sectional and non-random nature of SHG membership in the present study, the observed difference should not be interpreted as a causal effect of SHG participation.

**Table 4:** Comparison of socio-economic empowerment index.

Particulars	*N*	X̅	SD	*t*
Economic empowerment index				
Members	80	65.49	22.58	3.3**
Non- members	80	54.06	21.21	
Social empowerment index				
Members	80	69.40	08.92	25.59**
Non-SHG members	80	37.77	06.53	

**Significant at 1 per cent level of significance (*p* < 0.01), *Significant at 5 per cent level of significance (*p* < 0.05).

#### 3.3.2 Social empowerment index

The social empowerment indicators also showed substantial differences between the two groups (Supplementary Figures S3, S4). SHG members reported comparatively higher levels of self-confidence, social awareness and recognition, skills and access to facilities and services. For example, the supplementary figures show a considerably larger proportion of SHG members reporting higher levels of confidence and skills, whereas responses among non-SHG members were more concentrated in the lower or intermediate categories. The mean social empowerment index score was 69.40 ± 8.92 among SHG members, compared with 37.77 ± 6.53 among non-SHG members and the difference was statistically significant (*p* < 0.01) (Table 4). The substantially higher score among SHG members indicates a difference in the measured social empowerment dimension between the groups. Similar patterns have been reported by Kumari et al., 2026; Sahni et al. (2022) and Jadoun et al. (2021). Nevertheless, these findings represent group-level associations and should not be interpreted as evidence that SHG membership itself caused the observed differences.

### 3.4 Financial performance of SHGs members

The financial performance of the sampled SHGs was assessed using average savings, lending, outstanding amount, thrift-credit ratio, outstanding ratio and recovery ratio (Table 5). During the study period, the average savings amounted to ₹15,912.50, while average lending was ₹29,637.50. The average outstanding amount was ₹2,348.75. The thrift-credit ratio was 0.54, indicating that lending exceeded the reported savings base, while the outstanding ratio was 0.08. The average recovery ratio was 72.75%, indicating that nearly three-fourths of the reported loan demand, including principal and interest, had been recovered during the reference period. Overall, these indicators describe the financial performance of the sampled SHGs during the study period, particularly in terms of savings mobilisation, lending, outstanding credit and recovery. The observed recovery ratio was lower than the 80–98% range reported in earlier studies by Madheswaran and Dharmadhikary (2001), Devi and Jain (2011), and Ranjan and Tyagi (2015). These differences may be related to variations in the study period, loan structure, repayment conditions and institutional settings.

**Table 5:** Financial performance of the sample SHG.

Sr. No.	Particulars	Amount (₹)
1.	Average savings	15912.50
2.	Average lending	29637.50
3.	Average outstanding amount	2348.75
4.	Amount recovered	24562.50
5.	Demand raised (Principal amount + Interest)	33762.50
6.	Thrift credit ratio	00.54
7.	Outstanding ratio	00.08
8.	Average recovery ratio	72.75

### 3.5 Constraints faced by tribal women SHG members and non-SHG members in dairy management practices

The major constraints faced by tribal women in adopting dairy management practices were assessed separately for SHG and non-SHG members using Garrett’s ranking technique (Table 1). The ranking patterns showed that several constraints were common to both groups, while the relative importance of some constraints differed. For breeding management, the high cost of improved animal breeds was ranked first by both SHG members (62.36) and non-SHG members (61.21), followed by the non-availability of good crossbred animals or improved buffaloes. Lack of knowledge about artificial insemination ranked third in both groups, indicating that breeding-related costs and technical constraints were important concerns across the two groups. For feeding management, the highest-ranked constraint differed between the groups. The high cost of concentrates/mineral mixtures was ranked first by SHG members (64.00), whereas lack of green fodder during summer was the leading constraint among non-SHG members (64.58). Lack of knowledge about dry-fodder enrichment and balanced rations also appeared among the important constraints for both groups.

In healthcare management, the distant location of veterinary hospitals was the highest-ranked constraint among SHG members (62.00), while lack of knowledge about common contagious diseases was ranked first by non-SHG members (57.00). High medicine costs were also ranked among the major constraints by both groups. These findings indicate that both physical access to veterinary services and disease-related knowledge were important areas of concern. For milking management, the non-remunerative price of milk was the highest-ranked constraint for both SHG members (65.44) and non-SHG members (63.06). However, non-SHG members gave a relatively higher ranking to lack of knowledge about value-added dairy products, whereas SHG members ranked the non-existence of village-level milk producer cooperative societies as a more important constraint. Among general constraints, insufficient availability of loans was ranked first by SHG members (64.49), whereas lack of knowledge about animal insurance was the highest-ranked constraint among non-SHG members (59.50). Lack of information about government dairy development schemes was also reported as an important constraint by both groups. Overall, the findings show that cost, input availability, veterinary access, technical knowledge, milk prices, credit availability and information on insurance and government schemes were prominent constraints, although their relative rankings varied between SHG members and non-SHG members. Similar constraints related to dairy inputs, technical knowledge, veterinary services and institutional support have been reported in earlier studies (Bhawar et al., 2020; Ma et al., 2020; Brar et al., 2020; Jahan et al., 2024; Saurav et al., 2024). The differences in ranking should be interpreted as reported perceptions of constraints, rather than evidence that SHG membership caused differences in these constraints.

### 3.6 Limitations of the study

The findings of the study should be interpreted within the scope and methodological limitations of the research. First, the study was restricted to selected tribal areas of Sabarkantha and Aravalli districts, which limits the generalisability of the findings to other regions. Second, although multistage sampling was followed, the districts and talukas were purposively selected and the sample was limited to 160 respondents (80 SHG and 80 non-SHG members). Equal allocation was used to facilitate group comparison rather than population-proportionate representation. Third, SHG membership was not randomly assigned and respondents were not formally matched; therefore, pre-existing differences between the groups may have contributed to the observed differences. Although regression analysis was used to examine associations, residual confounding cannot be ruled out and the findings should not be interpreted as causal effects of SHG membership. Finally, the cross-sectional design limits the assessment of long-term changes, while self-reported information may be subject to recall or response bias. Future studies should consider larger probability-based samples, longitudinal designs and stronger matching or adjustment approaches to improve generalisability and causal interpretation.

## 4 Conclusion

This study provides a comparative assessment of dairy management practice adoption and socioeconomic empowerment among tribal women belonging to SHG and non-SHG households in selected areas of Sabarkantha and Aravalli districts of North Gujarat. The findings showed measurable differences in overall dairy management practice adoption between the two groups. The overall adoption score was 70.33 ± 13.83 among SHG members compared with 58.45 ± 5.11 among non-SHG members. Higher mean scores were observed among SHG members for feeding, animal shed, healthcare, milking and other management practices, whereas breeding management did not differ significantly between the groups. The study also identified differences in the measured economic and social empowerment dimensions. The mean Economic Empowerment Index was 65.49 ± 22.58 for SHG members and 54.06 ± 21.21 for non-SHG members, while the corresponding Social Empowerment Index scores were 69.40 ± 8.92 and 37.77 ± 6.53, respectively; both differences were statistically significant. Regression analysis further showed that landholding and herd size were statistically associated with dairy management adoption among SHG members, whereas dairy experience and annual income were associated with adoption among non-SHG members. The constraint analysis indicated that the cost of improved breeds, input costs, availability of green fodder, access to veterinary services, milk prices, credit availability and information on government schemes and animal insurance were among the important concerns reported by respondents. The financial assessment of the sampled SHGs also indicated average savings of ₹15,912.50, average lending of ₹29,637.50 and a recovery ratio of 72.75% during the study period. Overall, the findings highlight substantial variation in dairy management adoption and measured empowerment between SHG and non-SHG respondents in the selected study areas and identify socioeconomic characteristics and operational constraints relevant to dairy management. However, because the study was cross-sectional, the districts and talukas were purposively selected and SHG membership was neither randomly assigned nor formally matched, the observed differences should be interpreted as associations rather than causal effects of SHG membership. The findings are therefore most applicable to the selected tribal areas of Sabarkantha and Aravalli, while larger probability-based and longitudinal studies could provide stronger evidence for broader generalisation.

## Data Availability

The raw data supporting the conclusions of this article will be made available by the authors, without undue reservation.
