# Supplementary material for: Comparative assessment of dairy management practice adoption among SHG and non-SHG in tribal households of North Gujarat
Source: Front Sociol. 2026 Oct 22;11:1928015. doi: 10.3389/fsoc.2026.1928015 (PMC13735572; doi:10.3389/fsoc.2026.1928015)
Supplement: Supplementary file 1 [file Data_sheet_1.pdf]

Supplemental Information

Comparative Assessment of Dairy Management Practice Adoption among SHG and Non-SHG in Tribal Households of North Gujarat

Khushboo Kumari[1&2], Laxmi R. Dubey[2], Dilip R. Vahoniya[1*], Alvirakumari A. Rajwadi[3], Prakashkumar H. Patel[4], Mahin Sharif[5], Niraj Kumar[6], Samir Sen[7]

[1]International Agribusiness Management Institute, Anand Agricultural University, Anand, Gujarat

[2]College of Agribusiness Management, Sardar Krushinagar Dantiwada Agricultural University, Banaskantha, Gujarat

[3]College of Agriculture, Junagadh Agricultural University, Amreli, Gujarat

[4]Department of Commerce and Business Management, The Maharaja Sayajirao University of Baroda, Vadodara, Gujarat

[5]Department of Agricultural Economics, College of Agriculture, GKVK, University of Agricultural Sciences Bangalore

[6]Department of Soil and Water Conservation Engineering, Junagadh Agricultural University, Junagadh, Gujarat

[7]College of Food Processing Technology and Bio-Energy, Anand Agricultural University, Anand, Gujarat

***Corresponding author:** ksandilyaa@gmail.com

Table of contents

Tables/Figures	Page No.
Table S1	2
Table S2	2-3
Table S3	3-5
Table S4	5
Table S5	6
Table S6	7
Table S7	8
Fig. S1	9
Fig. S2	9-10
Fig. S3	11
Fig S4	11

Table S1: District, talukas and villages wise distribution of respondents

Districts	Selected Talukas	Selected villages	SHG members	SHG non-members	Total Members
Sabarkantha	Khedbrahma	Ponai	5	5	10
		Bhoori dhebar	5	5	10
		Lilavanta	5	5	10
		Khedva	5	5	10
	Vijaynagar	Parosa	5	5	10
		Kharibedi	5	5	10
		Navabhaga	5	5	10
		Navakhola	5	5	10
Aravalli	Bhiloda	Malasa	5	5	10
		Kishangadh	5	5	10
		Jumsar chaapra	5	5	10
		Bedasan	5	5	10
	Meghraj	Naranpur	5	5	10
		Paninar	5	5	10
		Dharola	5	5	10
		Ghorvada	5	5	10
		Total:	80	80	160

Table S2: The socio-economic profile of tribal women

Sr. No	Categories	Score	References
**1.1**	**Age**		
	Young (18 to 35 years)	1	National Adult Education Programme (NAEP), The Ministry of Education, Government of India
	Middle (36 to 50 years)	2	
	Old (>50 years)	3	
**1.2**	**Education**		
	Illiterate	1	Pandya and Pandya (2008)
	Primary level (1st to 8th std)	2	
	Secondary level (9th to 10th std)	3	
	Higher Secondary (11th to 12th)	4	
	Graduate / post-graduation (>12th)	5	
**1.3**	**Family size**		
	Small (up to 4 members)	1	Manjunath (2010), Manjunath (2014) and Sowjanya (2014)
	Medium (5 to 8 members)	2	
	Large (> 8 members)	3	
1.4	**Housing type**		
	Pucca	1	

	Semi-pucca	2	Manjunath (2010), Manjunath (2014), and Sowjanya (2014)
	Kutcha	3	
**1.5**	**Size of land holding**		
	Marginal (up to 1 ha)	1	Pandya and Pandya (2008)
	Small (1.01 to 2.00 ha)	2	
	Semi Medium (2.01 to 4.00 ha)	3	
	Medium (4.01 to 10.00 ha)	4	
	Large (> 10.00 ha)	5	
**1.6**	**Family occupation**		
	Dairy	1	Chaudhari (2006)
	Agriculture + Dairy	2	
	Dairy + Others	2	
	Agriculture + Dairy + Others	3	
**1.7**	**Experience in dairy farming**		
	Up to 5 years	1	Pandya and Pandya (2008)
	>5 to 10 years	2	
	>10 to 15 years	3	
**1.8**	**Herd size**		
	Small (1-2 Animals)	1	Savita (2004)
	Medium (3-4 Animals)	2	
	Big (>4 Animals)	3	
**1.9**	**Quantity of milk produce**		
	Low (Up to 5 L)	1	Raval and Chandawat (2011)
	Medium (6 to 10 L)	2	
	High (Above 10 L)	3	
**1.10**	**Annual income (RS)**		
	Low (Up to 60,000)	1	Manjunath (2010), Manjunath (2014), and Sowjanya (2014)
	Medium (60,001-1,20,000)	2	
	High (>1,20,000)	3	

Table S3: Extent of adoption of dairy management practices by tribal women: Indicate the improved dairy management practices adopted by you

Sr. No.	Dairy management practices	Extent of adoption		
		Fully	Partially	Never
**I**	**Adoption of breeding management practices**			
**1.**	Possession of improved breed of cows/ buffaloes for milch purpose			
**2.**	Keeping watch on oestrous cycle and heat symptoms of cow/ buffaloes.<br>Symptoms: Mucus discharge from vulva, start frequent urination, become restless and bellowing, mounting on another animal, allow other animal to mount, swelling of vagina *etc*.			
**3.**	Practising artificial insemination (AI)			

**4.**	Practising AI/ natural Service within 12-14 hours after onset of oestrous			
**5.**	Practising pregnancy diagnosis<br>If the animal does not show any symptom of oestrous cycle in next 18-24 days, then the pregnancy diagnosis should be done after 60-90 days of service/AI			
**II**	**Adoption of feeding management practices**			
**1.**	Growing green fodder for animals feeding such as: Sorghum, Maize *etc*.			
**2.**	Fodder utilization methods:			
	a. Chaffing the dry fodder at 2-3 cm in length			
	b. Soaking the chaffed dry fodder in water for 7-8hours			
	c. Salt water, Jaggery or Urea treatment of dry fodder			
	d. Silage or Haymaking			
**3.**	Providing daily requirement of production ration (day/animal):			
	a. Daily requirement of green fodder (30-35 kg)			
	b. Daily requirement of dry fodder (8-10kg)			
	c. Daily requirement of concentrates depending up on the milk production (2-4 kg)			
**4.**	Feeding colostrum:			
	a. Feeding colostrum to newly born calves within half an hour			
	b. Feeding colostrum to newly born calves at the rate of 10% of body weight for minimum 5 days			
**5.**	Providing 3-4 times clean drinking water to the animals daily			
**III**	**Adoption of animal shed management practices**			
**1.**	Head-to-head system/ tail-to-tail system of housing			
**2.**	Providing facilities of ideal byre (12-15 ft. height, 1" slope in legs, Gutter for easy flow of urine *etc*.)			
**3.**	Separate place for calf			
**4.**	Good ventilation and light facility			
**5.**	Maintaining cleanliness of animal byre Removing dung and urine from the byre frequently and keeping it dry			
**IV**	**Adoption of Healthcare management practices**			
**1.**	Daily washing of the animals (Grooming/bathing of animals)			
**2.**	Removing mucus from nose and mouth of			

	the calves just after birth			
**3.**	Practising deworming in calves for the prevention of parasitic diseases			
**4.**	Vaccination of animals timely and regularly against the contagious diseases like Hemorrhagic Septicemia, Black Quarter, Foot and Mouth disease			
**5.**	Segregating the diseased animals suffering from Contagious disease from healthy animals			
**6.**	Controlling the insect problems in animal shed (lice, bug, mosquitoes and flies. *etc*.)			
**V**	**Adoption of milking management practices**			
**1.**	Practising methods of milking:			
	a. Hand milking			
	b. Machine milking			
**2.**	hygienic practices, such as washing the udder and hands before milking			
**3.**	Using the clean/ washed utensils for milking and does not using these utensils for other purposes			
**4.**	Filtering of milk with a sieve or muslin cloth after milking			
**5.**	Milk sale record			
**VI**	**Adoption of other management practices**			
**1.**	Controlling the bad habits in animals such as Licking, Kicking, Fence breaking/jumping, Eating rope, clothes and plastics *etc.*			
**2.**	Practising the dry period in cow/buffaloes before 3 months of parturition			
**3.**	Record maintaining such as Breeding record, Animal feed/fodder record, Animal health record			
**4.**	Insurance of animals			

Table S4: Distribution of SHG members and non-members according to overall extent of adoption of dairy management practices

S. No.	Categories	SHG members	Non-SHG members
1	Low (<52.41)	7 (8.75)	9 (11.25)
2	Medium (52.41–76.36)	45 (56.25)	67 (83.75)
3	High (>76.36)	28 (35.00)	4 (5.00)
	Total	80 (100.00)	80 (100.00)

Mean = 64.39, SD = 11.98

Table S5: Score of economic empowerment index for SHG members (n=80)

Sr. No.	Economic indicators /scores	1	2	3	4	5	6
1.	**Household assets**	Below 42500 (08)	50000 (09)	57500 (13)	65000 (10)	72500 (15)	Above 72500 (25)
2.	**Household income**	Below 41000 (12)	59000 (16)	77000 (11)	95000 (10)	113000 (9)	Above 113000 (22)
3.	**Expenditure**	Below 37400 (09)	43000 (07)	48600 (15)	54200 (17)	59800 (07)	Above 59800 (25)
4.	**Savings**	Below 6800 (09)	7400 (15)	8200 (09)	9000 (04)	9800 (15)	Above 9800 (28)
5.	**Loan**	Below 12500 (33)	15000 (01)	17500 (17)	20000 (00)	22500 (08)	Above 22500 (21)
6.	**Housing type**	Kutcha (rent) (00)	Kutcha (own)(15)	Semi-pucca (rent) (00)	Semi-pucca (own) (27)	Pucca (rent) (00)	Pucca (own) (38)

Table S6: Score of economic empowerment index for SHG non-members (n=80)

Sr. No.	Economic indicators /scores	1	2	3	4	5	6
**1.**	**Household assets**	Below 42500 (18)	50000 (14)	57500 (16)	65000 (12)	72500 (9)	Above 72500 (11)
**2.**	**Household income**	Below 41000 (20)	59000 (18)	77000 (15)	95000 (10)	113000 (9)	Above 113000 (8)
**3.**	**Expenditure**	Below 37400 (15)	43000 (14)	48600 (17)	54200 (11)	59800 (8)	Above 59800 (15)
**4.**	**Savings**	Below 6800 (20)	7400 (18)	8200 (13)	9000 (7)	9800 (10)	Above 9800 (12)
**5.**	**Loan**	Below 12500 (36)	15000 (3)	17500 (14)	20000 (0)	22500 (9)	Above 22500 (18)
**6.**	**Housing type**	Kutcha (rent) (2)	Kutcha (own) (27)	Semi-pucca (rent) (0)	Semi-pucca (own) (24)	Pucca (rent) (0)	Pucca (own) (27)

Table S7: Score of social empowerment index

Sr. No.	Social indicators/ Score	Not at all	Very little	Somewhat	Much more	To a great extent
		1	2	3	4	5
**Self-confidence**						
**1.**	Confidence of financial crisis					
**2.**	Confidence in meeting official people					
**3.**	Physical mobility					
**Social awareness and recognition**						
**4.**	Social awareness					
**5.**	Respect in the family					
**6.**	Ability to take up social issues					
**Skills**		**Extremely poor**	**Poor**	**Satisfactory**	**Good**	**Excellent**
**7.**	Communication skills					
**8.**	Business skills					
**9.**	Financial skills					
**Facilities/services**						
**10.**	Medical facilities					
**11.**	Hygienic sanitation facilities					
**12.**	Drinking water supply					

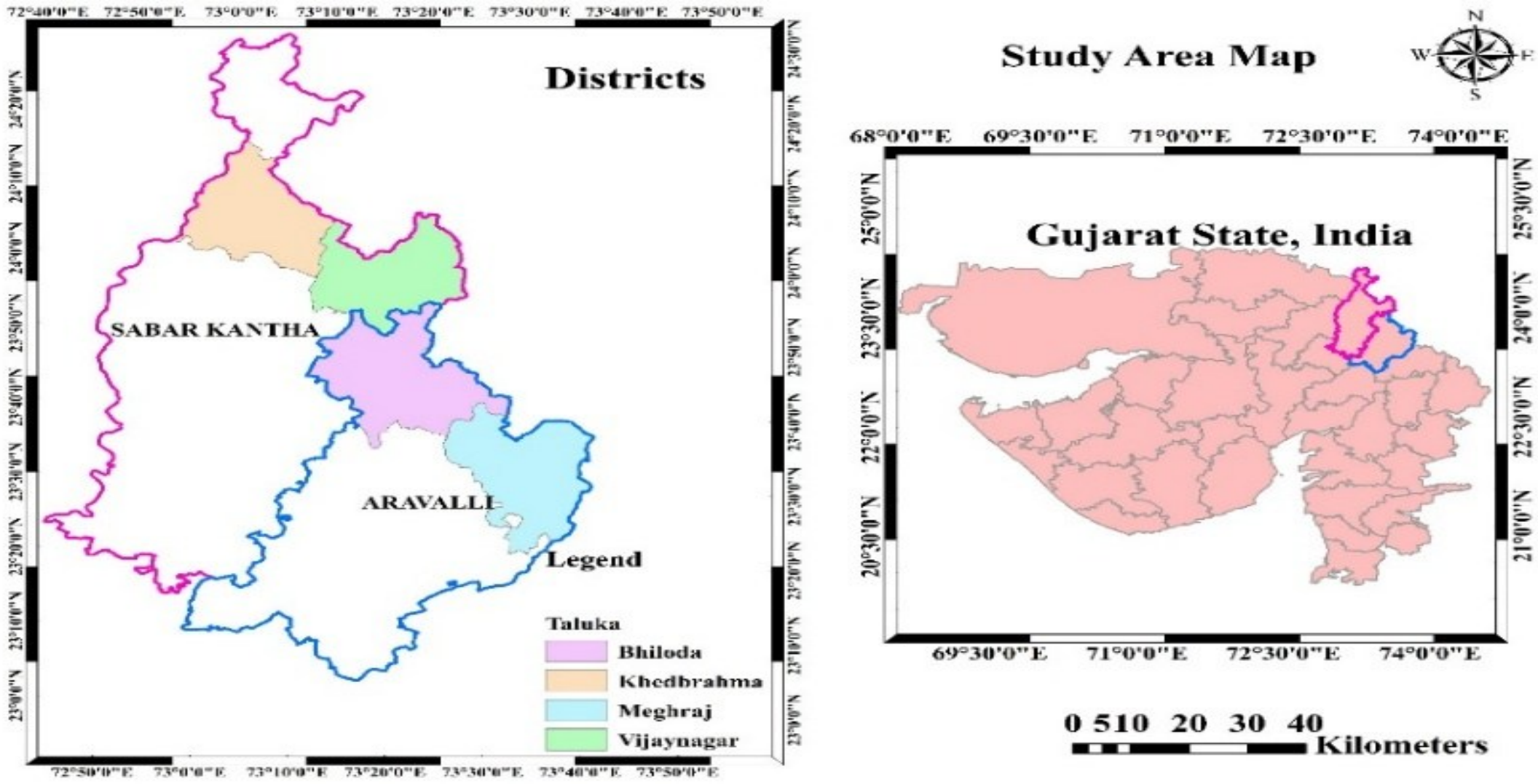

Figure S1: Map showing the study area

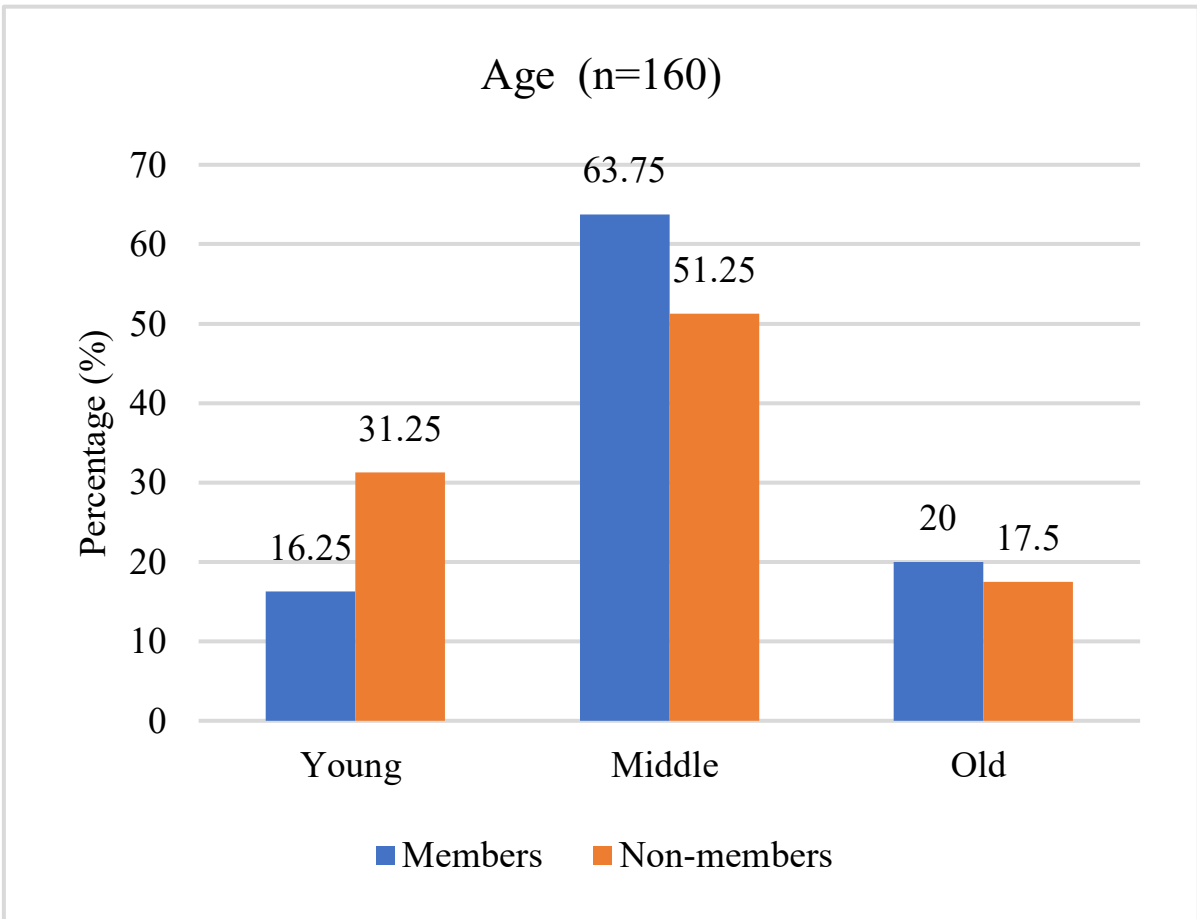

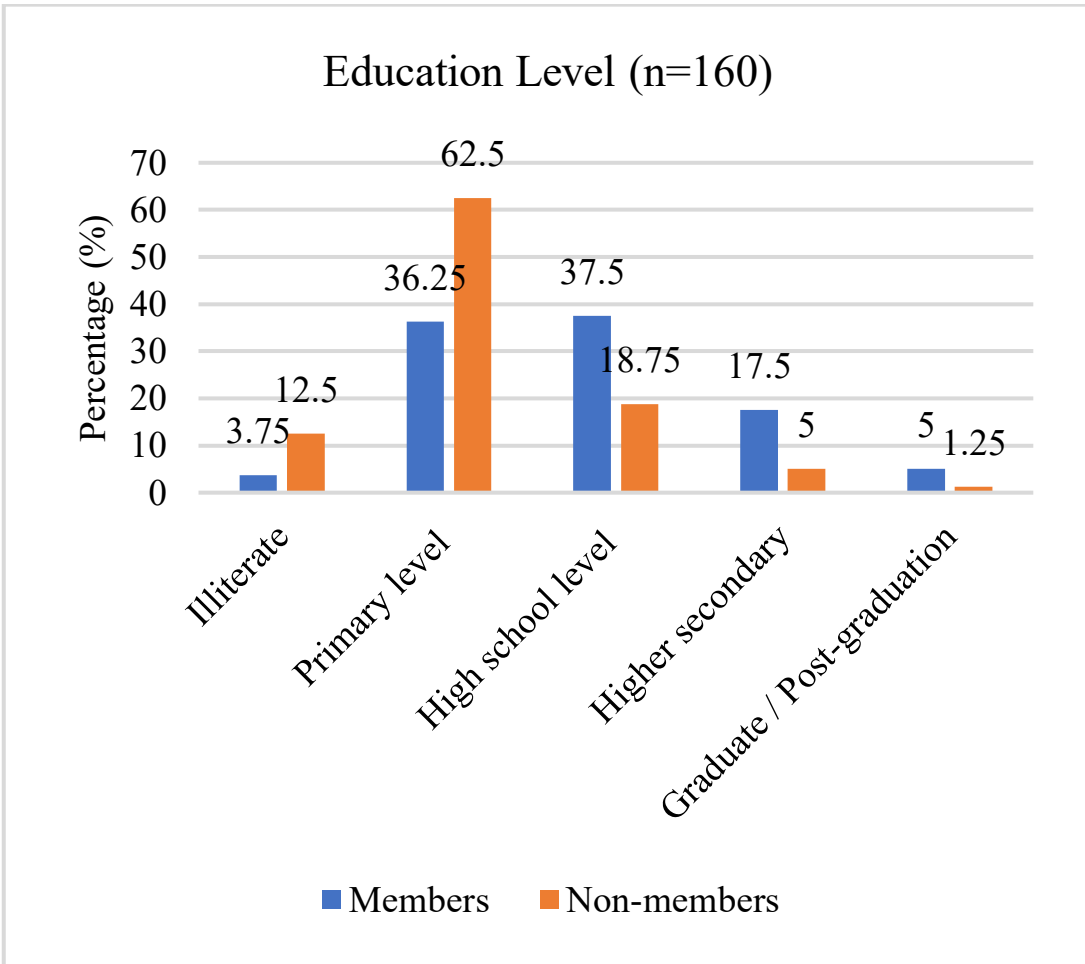

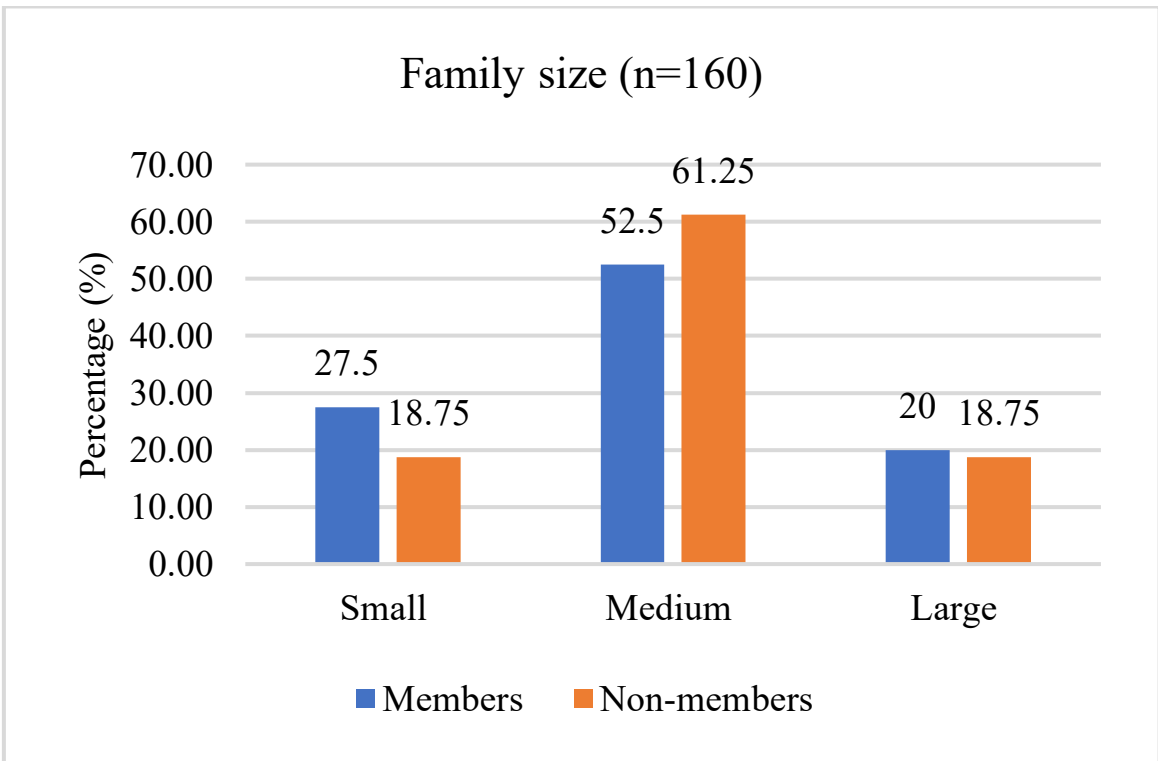

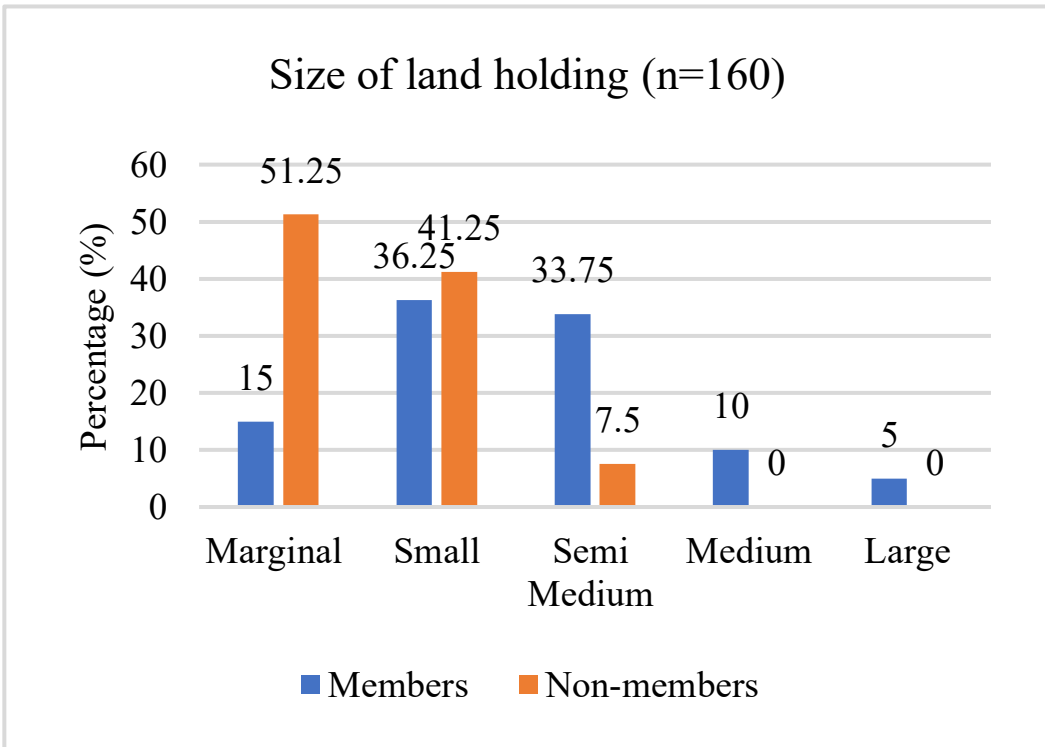

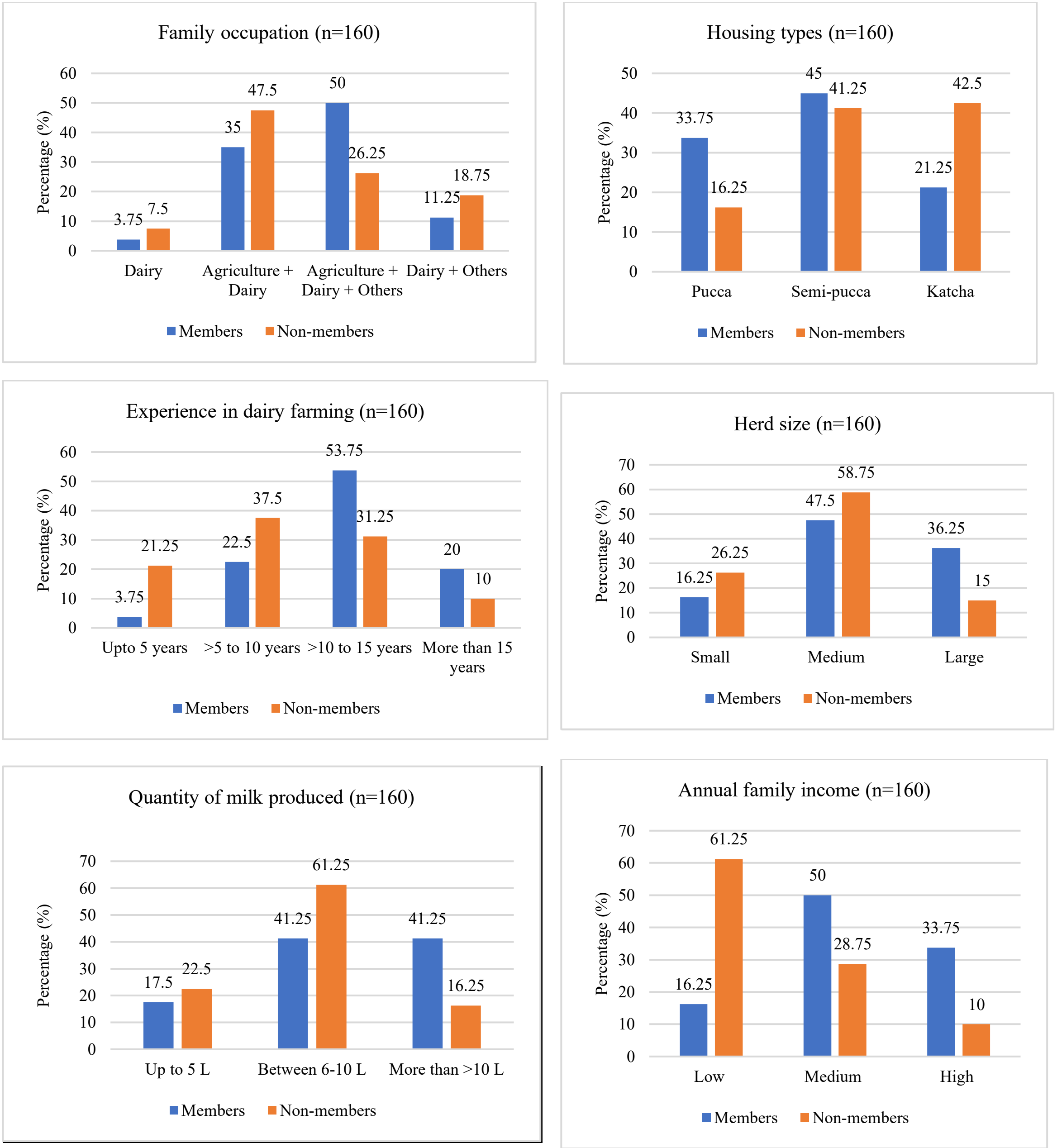

Figure S2: Graphical distribution of sample respondents according to socio-economic characteristics

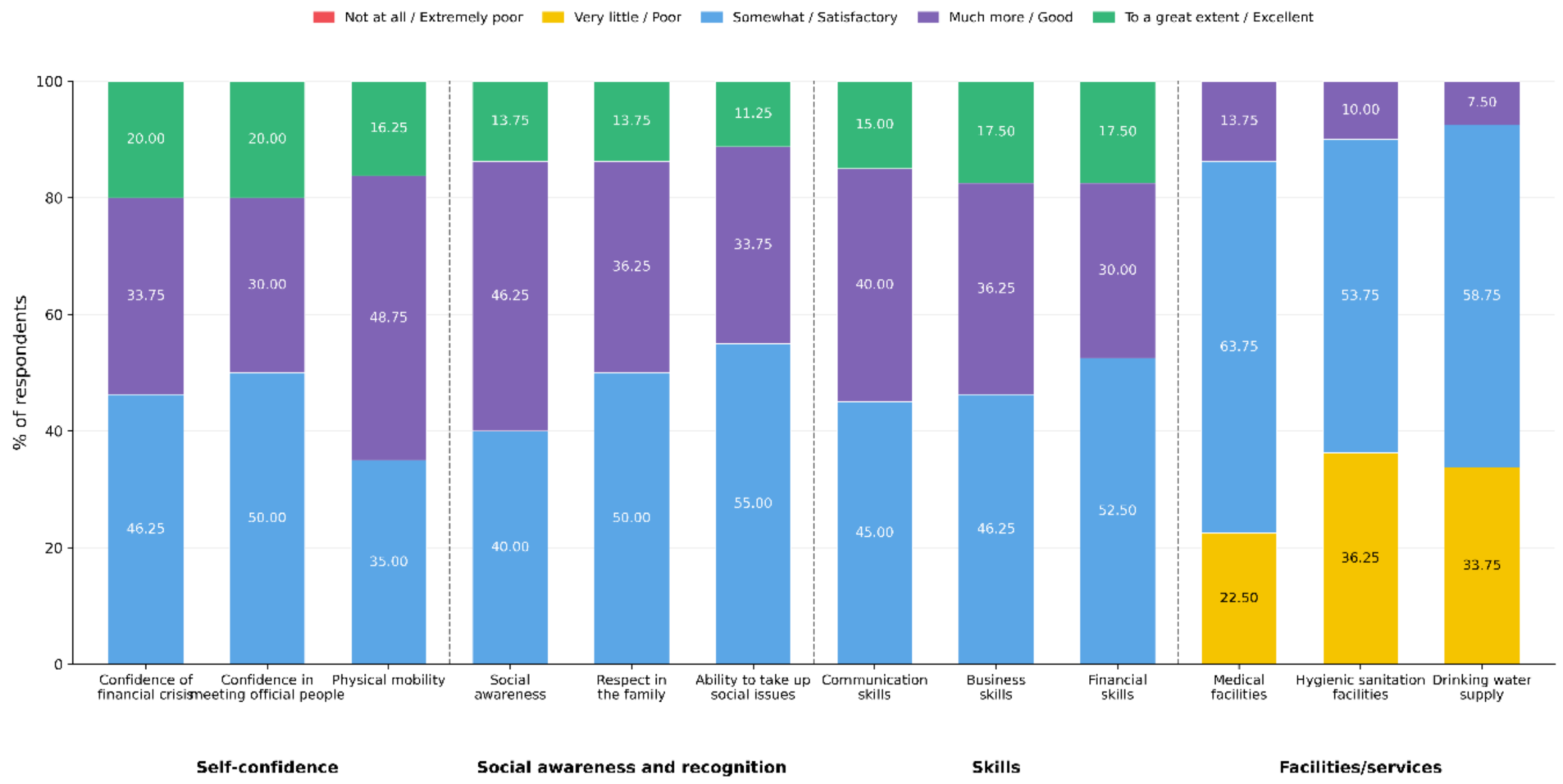

Figure S3: Score of social empowerment index for SHG members

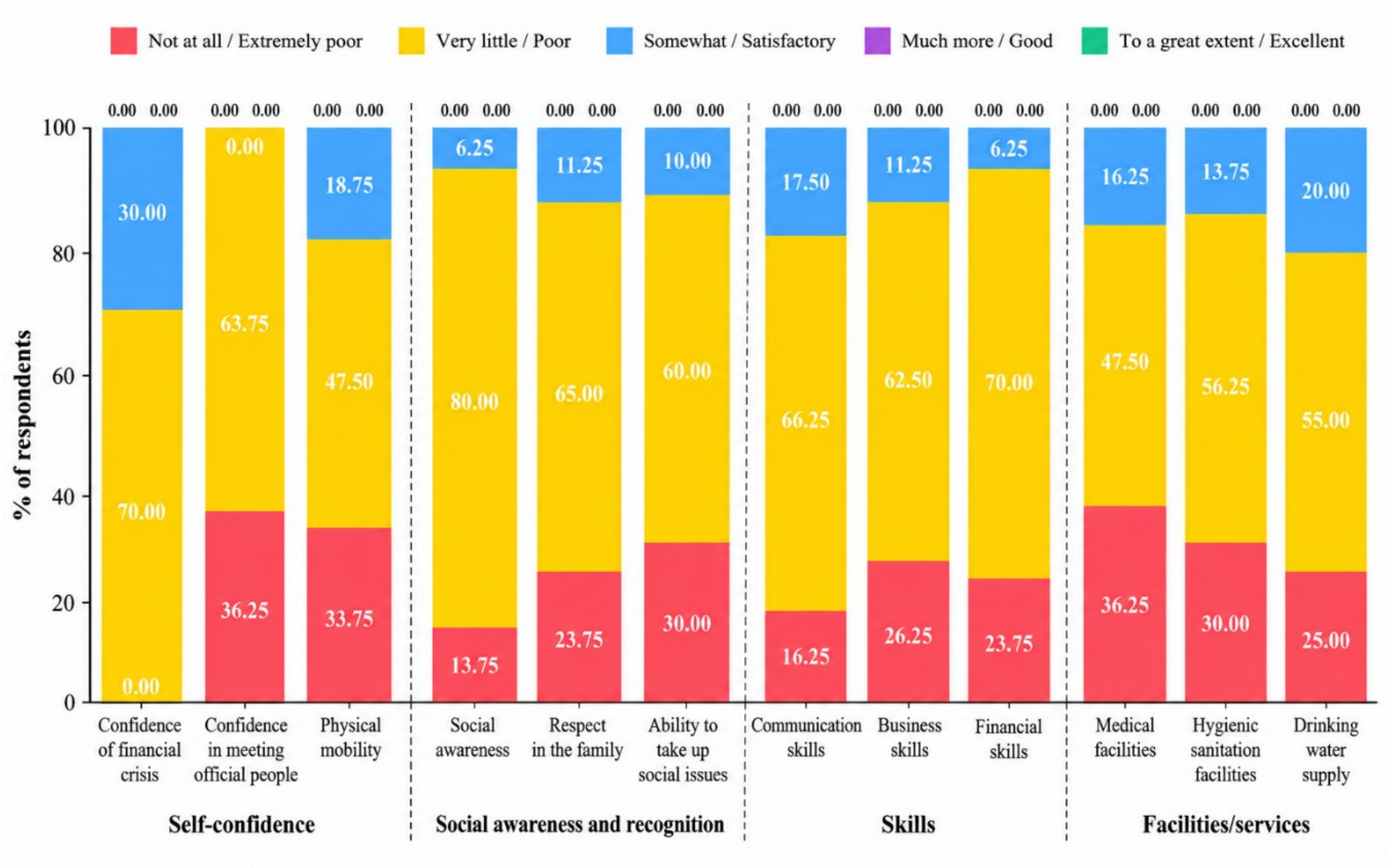

Figure S4: Score of social empowerment index for SHG non-members
